# Clinical characteristics and outcomes of *Mycobacterium tuberculosis *disease in adult patients with hematological malignancies

**DOI:** 10.1186/1471-2334-11-324

**Published:** 2011-11-23

**Authors:** Chien-Yuan Chen, Wang-Huei Sheng, Aristine Cheng, Woei Tsay, Shang-Yi Huang, Jih-Luh Tang, Yee-Chun Chen, Jaun-Yuan Wang, Hwei-Fang Tien, Shan-Chwen Chang

**Affiliations:** 1 Department of Internal Medicine, Division of Hematology, National Taiwan University Hospital, Taipei, Taiwan; 2 Department of Internal Medicine, Division of Infectious disease, National Taiwan University Hospital, Taipei, Taiwan; 3 Department of Internal Medicine, Division of Chest, National Taiwan University Hospital, Taipei, Taiwan; 4 Department of Internal Medicine, National Taiwan University Hospital, No. 7, Chung-Shan South Road, Taipei 100, Taiwan

**Keywords:** *Mycobacterium tuberculosis (TB)*, Hematological malignancy, Febrile neutropenia

## Abstract

**Background:**

Diseases caused by *Mycobacterium tuberculosis *(TB) among adult patients with hematological malignancies have rarely been investigated.

**Methods:**

Adult patients with hematological malignancies at National Taiwan University Hospital between 1996 and 2009 were retrospectively reviewed. Patients with positive serology for HIV were excluded. TB disease is diagnosed by positive culture(s) in the presence of compatible symptoms and signs. The demographics, laboratory and, microbiological features, were analyzed in the context of clinical outcomes.

**Results:**

Fifty-three of 2984 patients (1.78%) were diagnosed with TB disease. The estimated incidence was 120 per 100,000 adult patients with hematological malignancies. Patients with acute myeloid leukemia had a significantly higher incidence of TB disease than other subtypes of hematological malignancies (2.87% vs. 1.21%, *p *= 0.002, odds ratio, 2.40; 95% confidence interval, 1.39-4.41). Thirty-eight patients (72%) with non-disseminated pulmonary TB disease presented typically with mediastinal lymphadenopathy (53%), pleural effusion (47%) and fibrocalcific lesions (43%) on chest imaging. The 15 (28%) patients with extra-pulmonary disease had lower rates of defervescence within 72 h of empirical antimicrobial therapy (13% vs 45%, *p *= 0.03) and a higher 30-day in-hospital mortality (20% vs. 0%, *p *= 0.004) compared to those with disease confined to the lungs.

**Conclusions:**

TB disease is not uncommon among patients with hematological malignancies in Taiwan. Patients who received a diagnosis of extra-pulmonary TB suffered higher mortality than those with pulmonary TB alone. Clinicians should consider TB in the differential diagnoses of prolonged fever in patients with hematological malignancies, particularly in regions of high endemicity.

## Background

Although progress continues to be made with novel target therapy, chemotherapy remains the standard treatment in most patients with hematological malignancies. Side effects of chemotherapy include nausea, vomiting, mucositis, neutropenia and impaired humoral and cellular immunity. Febrile neutropenia is a common complication after chemotherapy; and infection is the leading cause of morbidity and mortality among patients with hematological malignancies. Bacteria and fungi are the major etiological agents of chemotherapy induced febrile neutropenia in patients with hematological malignancies [1,2]. *Mycobacterium tuberculosis *(TB) is a slow growing microbe that can cause disease in both healthy and immunocompromised persons. Yet TB disease in hematological patients has been rarely investigated [3-5].

The clinical manifestations of TB disease differ between cancer and non-cancer patients [3-5]. Cancer patients infected with TB frequently manifest more atypical clinical symptoms and signs [6,7]. The lung is still the most commonly involved site among cancer patients. However, extra-pulmonary tuberculosis appears not to be uncommon [5]. The incidence and mortality of TB disease are reportedly higher for cancer patients compared to otherwise healthy patients [6,7]. Ku et al. [8] and Cordonnier et al. [9] had previously studied TB disease in hematopoietic stem cell recipients. Since hematopoietic stem cell recipients usually receive intensive immunosuppressant regimens, they are at heightened risk of acquiring opportunistic infections. The diagnosis of TB disease is often difficult and delayed in hematopoietic stem cell recipients [8,9]. Delays in diagnosis of TB disease foreseeably could lead to fatal outcomes in patients with acute leukemia [10].

Taiwan is an endemic area for TB disease with an increasing trend of incidence over the past 40 years [11]. To define the role of TB disease in patients with hematological malignancies, we should improve the diagnostic accuracy and direct therapeutic strategy in late stages of febrile neutropenia in patients residing in regions of endemicity. In our previous epidemiological study, up to 60% of bloodstream isolates in febrile neutropenia consisted of gram negative pathogens and the leading gram negative pathogens were *Escherichia coli, Klebsiella pneumonia, Acinetobacter baumannii, Stenotrophomonas maltophilia*, and *Pseudomonas aeruginosa *[12]. How to differentiate between competing diagnoses of disease due to TB or aerobic bacteria (such as Enterobacteriaceae) in hematological patients with febrile neutropenia is elemental in clinical practice. In this study, we retrospectively reviewed hematological patients with TB disease in a single university hospital in Taiwan to investigate the clinical characteristics and outcomes of TB disease in hematological patients with febrile neutropenia.

## Methods

### Patients and hospital setting

National Taiwan University Hospital (NTUH) is a 2600-bed teaching hospital in northern Taiwan that provides both primary and tertiary care. Lymphoma, acute myeloid leukemia and multiple myeloma were the most common diagnosis of hematological malignancies [1,12]. All adult (age ≧ 18 years) hematological patients enrolled in this retrospective study were patients admitted between January 1996 and December 2009. Patients with hematological malignancies who were HIV-positive were excluded. Demographic features, hematological disease status, underlying medical diseases, laboratory and microbiological data, and outcomes were collected and analyzed retrospectively. The incidence of TB disease in patients with various hematological malignancies was calculated. This research conformed to the Helsinki Declaration and local legislation, and was approved by the local ethics committee.

### Definition and diagnosis of *M. tuberculosis*

Definite diagnosis of *M. tuberculosis *disease was made based on clinical symptoms and signs and positive culture(s) for *M. tuberculosis *from sputum and/or tissue. To prevent inclusion of non-tuberculosis mycobacterial cases, patients with clinical suspicion of *M. tuberculosis *infections but without documented culture results were excluded from this study. Pathological findings of caseous necrosis, Langerhan giant cell and acid-fast stain positive were in themselves insufficient as criteria for inclusion. Synchronous infection was defined as a diagnosis of TB disease within 3 months of hematological disease diagnosis [5,13]. Readings of plain chest radiographs for all patients at admission and diagnosis of TB infection were reviewed by radiologists.

### Statistical analysis

Categorical variables were compared using the chi-squared test. Continuous variables were compared using the Student's t-test or ANOVA test. The significance level was set at 0.05 and all *p *values were two-tailed. The survival stratified by pulmonary and extra-pulmonary tuberculous was analyzed by Kaplan-Meier method and log-rank test. All statistical analyses were performed using SPSS 18.0 for Windows (SPSS, Chicago, IL, USA).

## Results

### Epidemiology of *M. tuberculosis *disease

**Figure 1:**
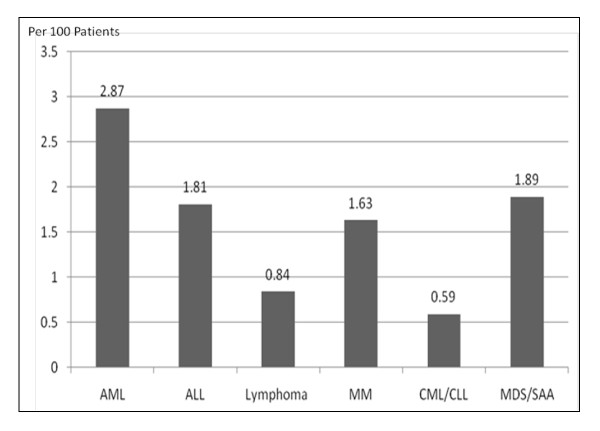
**Percentage of tuberculosis in patients with hematological malignancy between 1996 and 2009**.

Between 1996 and 2009, a total of 2984 patients were admitted to NTUH with hematological malignancies, including 1011 patients diagnosed with acute myeloid leukemia (AML), 276 patients with acute lymphoblastic leukemia (ALL), 956 patients with lymphoma, 307 patients with multiple myeloma (MM), 169 patients with chronic myeloid leukemia (CML) or lymphoid leukemia (CLL), and 265 patients with myelodysplastic syndrome (MDS) or severe aplastic anemia (SAA). Of these, 53 patients (37 males and 16 females) with a median age of 58 years (range, 27-86 years) were diagnosed with TB disease. The overall incidence of TB disease in patients with hematological malignancies during the study period was 1.78%. The numbers of TB cases among each subtype of hematological malignancies were in descending order as follows: AML, *n = *29 (2.87%); ALL, *n = *5 (1.81%); lymphoma, *n = *8 (0.84%); MM, *n = *5 (1.63%); CML/CLL, *n = *1 (0.59%); and MDS/SAA, *n = *5 (1.89%) (Figure 1). Patients with AML were more likely to develop TB disease than patients with other hematological malignancies (2.87% vs. 1.12%, *p *= 0.002; odds ratio, 2.40; 95% confidence interval, 1.39-4.41).

### Clinical and laboratory characteristics of patients with *M. tuberculosis *disease

The clinical manifestations and laboratory results of 53 patients with TB disease are shown in Table 1. Twenty-six (49%) patients were diagnosed with TB disease at the time of febrile neutropenia, while 27 (51%) patients were diagnosed (including 5 patients without fever at diagnosis) with a non-neutropenic status. Patients diagnosed during febrile neutropenia were more likely to be patients with AML as opposed to other hematological subtypes (19 of 29 patients, 66% vs. 7 of 24 patients, 29%, *p *= 0.013) and were synchronously diagnosed with TB disease and hematological malignancies (54% vs. 11%, *p *= 0.001) more frequently than non-neutropenic counterparts.

**Table 1:** Clinical and laboratory manifestations of 53 haematological patients with *Mycobacterium tuberculosis *(TB) infection

	Total (*n *= 53)	Neutropenia			Sites of tuberculosis		
		
		No (*n *= 27)	Yes (*n *= 26)	*P *value	Pulmonary TB only (*n *= 38)	Extra-pulmonary involvement^e ^(*n *= 15)	*P *value
Elderly				0.28			0.24

Age more than 60 year	25	15	10		20	5	

Age less than 60 year	28	12	16		18	10	

Gender				0.56			0.34

Male	37	20	17		28	9	

Female	16	7	9		10	6	

Underlying Diabetes mellitus				0.67			0.66

Yes	6	4	2		5	1	

No	47	23	24		33	14	

Hematological Disease				0.013			0.76

Acute myeloid leukemia	29	10	19		20	9	

Non-Acute myeloid leukemia	24	17	7		18	6	

Diagnosis of tuberculosis and malignancy				0.001			0.33

Synchronous^a^	17	3	14		14	3	

Non-synchronous	36	24	12		24	12	

Allogeneic stem cell transplantation				0.19			> 0.99

Yes	6	5	1		4	2	

No	47	25	22		34	13	

Defeverence within 72 hours^b^				0.55			0.030

Yes	17	9	8		15	2	

No	31	13	18		18	13	

Extra-pulmonary tuberculosis				> 0.99			

Yes	38	19	19				

No	15	8	7				

Laboratory data^c^							

Absolute neutrophil count (/uL) at fever	1970	3587	198	0.019	2174	1100	0.87

Absolute neutrophil count (/uL) at nadir	39	761	17	< 0.001	39	39	0.59

Albumin (g/dL)	3.60	3.48	3.70	0.40	3.50	3.6	0.80

Serum alanine aminotransferase (IU/L)	30	26	34	0.67	34	27	0.44

Alkaline phosphatase (IU/L)	174	207	141	0.06	175	153	0.52

Lactate dehydrogenase (IU/L)	509	574	440	0.96	509	509	0.58

C-reactive protein (mg/dL)	5.42	5.43	5.16	0.60	5.59	4.73	0.48

Treatment^d^							

Standard therapy	39	20	19	> 0.99	31	8	0.048

Fluoroquinolone based therapy	9	4	5		4	5	

Outcome (30 days)							

Death	3	1	2	0.61	0	3	0.019

Alive	50	26	24		38	12	

^a^Synchronous define as diagnosis of tuberculosis within 3 months of hematological malignancy

^b^Five patients with pulmonary tuberculosis did not present with fever

^c^Median Value

^d^48 patients received anti-tuberculosis treatment

^e^Five of the 15 patients (33.3%) with extra-pulmonary tuberculosis (lymph node, 2; liver aspirates, 1; urine, 1; and blood, 1) also had documented pulmonary tuberculosis

**Figure 2:**
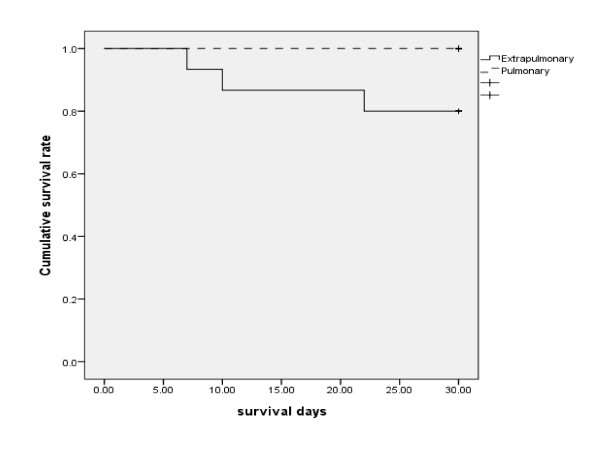
**30-day mortality between pulmonary and extra-pulmonary tuberculosis (*p *= 0.004)**.

Sputum acid-fast stains and cultures were performed in all 53 patients (median 3 sets, ranged 1-14). Of the 43 patients diagnosed with pulmonary tuberculosis, nine patients (21%) had smear-positive TB (both positive for acid-fast bacilli (AFB) in sputum smear and culture positive for TB); the remaining 34 patients were smear-negative. Fifteen of the 53 patients (28%) had extra-pulmonary disease, with positive cultures from lymph nodes (*n = *4), liver aspirates (*n = *3), synovial fluid (*n = *2), ascites (*n = *2), urine (*n = *2), abdominal wall abscess (*n = *1), and blood (*n = *1). Five of the 15 patients (33.3%) with extra-pulmonary tuberculosis (lymph nodes, 2; liver aspirate, 1; urine, 1; and blood, 1) also had concomitant pulmonary involvement. The characteristics of pulmonary only versus extra-pulmonary disease were similar in terms of absolute neutrophil count (2174 per uL vs. 1100 per uL, *p = *0.76) and hematological malignancies subtypes (AML subtype: 31% vs. non-AML subtype: 25%, *p *= 0.87). The median duration of fever in patients with extra-pulmonary tuberculosis was longer than those with disease confined to the lungs (15 days vs 9 days, *p *= 0.025). In addition, patients with extra-pulmonary TB had a higher 30-day mortality (*p *= 0.004, Figure 2) and were less likely to defervesce within 72 h after commencement of broad-spectrum antibiotic therapy (13% vs 45%, *p *= 0.03). (Table 1) There were no other significant predictive features to distinguish isolated pulmonary from extra-pulmonary TB disease.

### Drug resistance of *M. tuberculosis *and treatment outcomes

Nine of the 53 different TB isolates (17.0%) from each patient showed drug-resistance, including one isolate (1.9%) resistant to both isoniazid and rifampin (multi-drug resistant TB). The drug resistance percentages were as stated for isoniazid (*n *= 5, 9.4%), streptomycin (*n *= 4, 7.5%), rifampin (*n *= 1, 1.9%) and ethambutol (*n *= 1, 1.9%).

Forty-eight (91%) patients received anti-tuberculosis treatment during hospitalization; 39 (81%) patients received standard anti-tuberculosis treatment (isoniazide, rifampin, ethambutol, and pyrazinamide) and 9 (19%) patients received fluoroquinolone based anti-tuberculosis therapy. Patients with extra-pulmonary tuberculosis had a higher rate of fluoroquinolone-based therapy (38% vs. 11%, *p *= 0.048). No differences in the timing of anti-tuberculous therapy initiation in relation to the date of culture were observed between patients with neutropenia and those without (median interval, 20 days vs. 22 days, *p *= 0.52), nor between patients with only pulmonary disease versus extra-pulmonary disease (median interval, 22 days vs. 22 days, *p *= 0.31).

### Radiological characteristics of pulmonary tuberculosis

The radiological characteristics of 43 patients with pulmonary tuberculosis are shown in Table 2. The chest plain films at admission and at diagnosis of pulmonary tuberculosis were evaluated. Twenty-nine (67%) patients had chest computer tomographic images at diagnosis of pulmonary tuberculosis. Typical findings in descending order were mediastinal lymphadenopathy in 23 (53%) patients, pleural effusions in 20 patients (47%) and fibrocalcific parenchymal lesions in 16 patients (43%). Computer tomography (CT) significantly detected more mediastinal lymphadenopathy (*p *< 0.001) compared to plain chest radiography.

**Table 2:** Radiological characteristics of 43 patients with pulmonary tuberculosis

Radiological findings	Total (*n *= 43) (%)	Chest Plain film only (*n *= 14)	Chest CT scan (*n *= 29)	*P *value
Mediastinal and/or hilar lymphadenopathy	23 (53)	1	22	< 0.001

Pleural effusion	20 (47)	5	15	0.35

Fibrocalcified lesion	14 (43)	2	12	0.095

Nodules	16 (37)	4	12	0.51

Ground glass	6 (14)	0	6	0.16

Pericardial effusion	6 (14)	0	6	0.16

Interstitial pattern	2 (7)	1	1	> 0.99

Cavitation	2 (7)	0	2	> 0.99

CT, computed tomography

## Discussion

The increased utilization of anti-neoplastic agents in the treatment of hematological malignancies is associated with an increase of endemic opportunistic infections, for example, tuberculosis. Neutropenia, impaired humoral and cellular immunity are frequent complications in patients with hematological malignancies. The symptoms and signs of TB disease in patients with hematological malignancies may thus differ from the general population [3,5,14,15]. However, the epidemiology, clinical manifestations, and outcomes are mostly undetermined. In Taiwan, estimates of the incidence of TB disease ranged from 75 per 100,000 population to 62 per 100,000 population between 2002 and 2008 [16]. In this study, we estimated the incidence of TB disease to be approximately 120 per 100,000 for patients with hematological malignancies; namely an increase by two-fold above the general population. Similarly, Libshitz et al. had reported the incidence of TB disease to be 90 per 100,000 in cancer patients, a rate nine times greater than the general population in the United States [13]. These results show that TB disease is not uncommon among patients with hematological malignancies, especially in a tuberculosis endemic area.

The timing of tuberculosis reactivation or development in patients with hematological malignancies is not clear. Since 43% patients had fibrocalcified lesions on the chest CT, we infer that most patients might have had previous TB infection before febrile neutropenia. Reactivation of tuberculosis in patients with hematological malignancies could result from impaired immunity by underlying hematological malignancies and/or chemotherapy induced immunosuppression. The definite timing of patients developed active tuberculosis disease needs further prospective investigation.

Stefan et al. reported that acute lymphoblastic leukemia was the most common malignancy in children with tuberculosis [14]. Steroid and lymphoid malignancy both impair cellular immunity and potentially increase the risk of tuberculosis disease. However, Mishra et al. reported that patients with AML rather than lymphoma had a higher rate of *M. tuberculosis *disease [15]. Our results support the observation that adult AML patients might have a relatively higher rate of TB disease than other types of hematologic malignancies. Since neutrophil counts were unknown in previous studies [14,15], we discovered that tuberculosis in AML patients is often associated with significant febrile neutropenia compared to non-AML patients (66% vs. 29%, *p *= 0.013). The risk of acquiring TB disease may correlate partly with the patient's absolute neutrophil counts, but this association may not be uniform across different types of hematological malignancies. Recent research on human neutrophil peptides has highlighted their bactericidal action against TB and suggested that neutrophils may play a more important defensive role in tuberculosis [17]. Neutrophil could mediate innate immunity against mycobacterium. In an adult tuberculosis cohort, risk of TB disease was inversely and independently associated with peripheral blood neutrophil counts in patients diagnosed with pulmonary tuberculosis [18]. This result was consistent with our study supporting the important role of neutrophils in the defense against TB infection. TB disease might be evaluated as an important differential diagnosis for patients with hematological malignancies suffering from febrile neutropenia in a tuberculosis endemic area.

Extra-pulmonary presentation of TB disease is common in patients with hematological malignancies, ranging from 16% to 78% for all kinds of TB disease [5,13,19]. We observed that 28% of hematological patients with TB disease had extra-pulmonary manifestations, in line with prior studies [5,13,19]. In this study, we found that patients with extrapulmonary tuberculosis had prolonged fever associated with worse outcomes. Cytokines play a role in host defence of tuberculosis [20]. However, previous study revealed cytokine levels did not correlate with localization of pulmonary and extrapulmonary tuberculosis [20]. It might be possible that extrapulmonary TB represented disseminated disease and delayed diagnosis due to variable extrapulmonary manifestations in the patients with hematological malignancies contributed to excess mortality [21-23]. Extra-pulmonary tuberculosis often presented with various symptoms and signs, such as hepatosplenic tuberculosis, which may also mimic chronic disseminated candidiasis [24,25]. Diagnostic procedures in hematological patients are impeded by bleeding tendencies and neutropenic status, thus the diagnosis of extra-pulmonary tuberculosis could be delayed because of a lack of tissue proof and cultures. Thus, in hematological patients with prolonged fever without definite etiology, tuberculosis might be one of the differential diagnoses in the clinical practice of hemato-oncology.

Pulmonary infiltration is a frequent presentation of infectious diseases in cancer patients [13]. Although nonspecific pneumonia is one of the radiographic findings of pulmonary tuberculosis, it is not helpful for diagnosis. In this study, most patients with hematological malignancies and pulmonary tuberculosis showed mediastinal lymphadenopathy, pleural effusions, and fibrocalcified lesions. From this, reactivation of tuberculosis after immunocompromised status by hematological malignancies and/or chemotherapy associated neutropenia is highly suspected. Andreu et al. [26] reported that lymphadenopathy is the most characteristic radiological feature in tuberculosis. In enhanced chest CT, hilar and mediastinal nodes with a central hypodense area supported the diagnosis [26]. However, only one patient had the typical presentation of mediastinal lymph node with central hypodense lesion. This characteristic image finding is not sensitive enough to predict pulmonary tuberculosis. Chest CT scan revealed mediastinal lymphadenopathy in half of the patients with pulmonary tuberculosis, which could be a better predictor of culture positive tuberculosis. Computer tomography was significantly better at discovering mediastinal lymphadenopathy (*p *< 0.001) than plain chest radiography. Hence chest CT might be a more valuable tool for diagnosing pulmonary TB in hematological patients with clinical respiratory symptoms/signs after chemotherapy,

Severe neutropenia produces relatively less tissue inflammatory response after chemotherapy in hematological patients [27]. The atypical clinical presentations of TB disease in hematological patients makes a difficult diagnosis in the context of coexistent disease even more difficult [28]. By this token, we suggest that TB should always be considered even as a remote possibility so that unusual symptoms and signs are elicited, followed by at least three sputum examinations. A plain chest film to screen for tuberculosis in cancer patients with febrile neutropenia is recommended by Taiwanese guidelines [29]. However, for those patients without a definite diagnosis from chest plain films and ongoing pulmonary symptoms, we suggest chest tomography to be an appropriate modality for clinical diagnosis of TB disease in regions of high endemicity.

Critically ill tuberculosis patients may have symptoms of acute respiratory distress syndrome, disseminated intravascular coagulopathy, or infrequently septic shock [30,31]. The search for causative pathogens in febrile neutropenia is still a challenge in current clinical practices. According to this study, clinicians should remain alert to the differential diagnosis of tuberculosis in hematological patients with prolonged fever of unknown etiology.

This study had the following limitations. First, only hematological patients with proven tuberculosis were enrolled, and some patients with low mycobacterium burden might be excluded. Second, tissues biopsy and cultures were not available for all hematological patients because of bleeding tendency and neutropenic status. Third, this study was a retrospective cohort; some limitations such as concurrent infection in patients with febrile neutropenia cannot be completely excluded. Further large scale study should be conducted to confirm the findings based on our limited case numbers.

## Conclusions

In conclusion, infections caused by *M. tuberculosis *are not uncommon among patients with hematological malignancies in countries with a high incidence of tuberculosis. Tuberculosis causes significant morbidity and mortality in patients with various hematological malignancies and in recipients of hematopoietic stem cell transplants. Patients with extra-pulmonary tuberculosis had significantly higher 30-day mortality than those with pulmonary disease alone. Clinicians may choose to evaluate tuberculosis as a differential diagnosis for prolonged fever in patients with hematological malignancies in endemic areas.

## Competing interests

The authors declare that they have no competing interests.

## Authors' contributions

CYC, WHS, and AC designed, conducted the study and wrote manuscript. YCC, WT, JLT, SYH, JYW, SCC, HFT recruit patients and provide patients care. All authors read and approved the final manuscript.

## Pre-publication history

The pre-publication history for this paper can be accessed here:

http://www.biomedcentral.com/1471-2334/11/324/prepub

## References

[B1] Chen CY Tang JL Hsueh PR Yao M Huang SY Chen YC Chen YC Shen MC Wang CH Tsai W Chang SC Tien HF Luh KT Trends and antimicrobial resistance of pathogens causing bloodstream infections among febrile neutropenic adults with hematological malignancy J Formos Med Assoc 2004 103 526 532 15318274

[B2] Narita M Polymerase chain reaction for diagnosis of infectious diseases Acta Paediatr Jpn 1993 35 89 97 8503277 10.1111/j.1442-200x.1993.tb03015.x

[B3] Gupta A Singh M Singh H Kumar L Sharma A Bakhshi S Raina V Thulkar S Infections in acute myeloid leukemia: an analysis of 382 febrile episodes Med Oncol 2010 27 1037 45 10.1007/s12032-009-9330-9 19830601

[B4] Shamsi TS Irfan M Ansari SH Farzana T Khalid MZ Panjwani VK Baig MI Shakoor N Allogeneic peripheral blood stem cell transplantation in patients with haematological malignancies J Coll Physicians Surg Pak 2004 14 522 526 15353134

[B5] Kaplan MH Armstrong D Rosen P Tuberculosis complicating neoplastic disease. A review of 201 cases Cancer 1974 33 850 858 10.1002/1097-0142(197403)33:3<850::AID-CNCR2820330334>3.0.CO;2-H 4592905

[B6] Maartens G Wilkinson RJ Tuberculosis Lancet 2007 370 2030 2043 10.1016/S0140-6736(07)61262-8 17719083

[B7] Kobashi Y Mouri K Yagi S Obase Y Miyashita N Okimoto N Matsushima T Oka M Clinical features of immunocompromised and nonimmunocompromised patients with pulmonary tuberculosis J Infect Chemother 2007 13 405 410 10.1007/s10156-007-0558-z 18095090

[B8] Ku SC Tang JL Hsueh PR Luh KT Yu CJ Yang PC Pulmonary tuberculosis in allogeneic hematopoietic stem cell transplantation Bone Marrow Transplant 2001 27 1293 1297 10.1038/sj.bmt.1703092 11548848

[B9] Cordonnier C Martino R Trabasso P Held TK Akan H Ward MS Fabian K Ullmann AJ Wulffraat N Ljungman P Alessandrino EP Pretnar J Gmür J Varela R Vitek A Sica S Rovira M European Blood and Marrow Transplant Group Infectious Diseases Working Party Mycobacterial infection: a difficult and late diagnosis in stem cell transplant recipients Clin Infect Dis 2004 38 1229 1236 10.1086/383307 15127333

[B10] Ker CC Hung CC Sheng WH Chang SC Luh KT Fatal mycobacteremia caused by Mycobacterium tuberculosis in a patient with acute leukemia Leukemia 1999 13 646 647 10.1038/sj.leu.2401380 10214876

[B11] Yu MC Bai KJ Chang JH Lee CN Age transition of tuberculosis patients in Taiwan, 1957-2001 J Formos Med Assoc 2006 105 25 30 10.1016/S0929-6646(09)60105-4 16440067

[B12] Chen CY Tsay W Tang JL Tien HF Chen YC Chang SC Hsueh PR Epidemiology of bloodstream infections in patients with haematological malignancies with and without neutropenia Epidemiol Infect 2010 138 1044 1051 10.1017/S0950268809991208 19941686

[B13] Libshitz HI Pannu HK Elting LS Cooksley CD Tuberculosis in cancer patients: an update J Thorac Imaging 1997 12 41 46 10.1097/00005382-199701000-00006 8989758

[B14] Stefan DC Kruis AL Schaaf HS Wessels G Tuberculosis in oncology patients Ann Trop Paediatr 2008 28 111 116 10.1179/146532808X302125 18510820

[B15] Mishra P Kumar R Mahapatra M Sharma S Dixit A Chaterjee T Choudhry DR Saxena R Choudhry VP Tuberculosis in acute leukemia: a clinico-hematological profile Hematology 2006 11 335 340 10.1080/10245330600915818 17607583

[B16] Lo HY Chou P Yang SL Lee CY Kuo HS Trends in tuberculosis in Taiwan, 2002-2008 J Formos Med Assoc 2011 110 501 510 10.1016/S0929-6646(11)60076-4 21783019 PMC7134926

[B17] Fu LM The potential of human neutrophil peptides in tuberculosis therapy Int J Tuberc Lung Dis 2003 7 1027 1032 14598960

[B18] Martineau AR Newton SM Wilkinson KA Kampmann B Hall BM Nawroly N Packe GE Davidson RN Griffiths CJ Wilkinson RJ Neutrophil-mediated innate immune resistance to mycobacteria J Clin Invest 2007 117 1988 1994 10.1172/JCI31097 17607367 PMC1904316

[B19] Leung WH Tsang SF Chim CS Extrapulmonary tuberculous abscess in chronic lymphocytic leukaemia (CLL) treated with fludarabine: case report and review of literature Am J Hematol 2005 79 246 247 10.1002/ajh.20322 15981223

[B20] Verbon A Juffermans N Van Deventer SJ Speelman P Van Deutekom H Van Der Poll T Serum concentrations of cytokines in patients with active tuberculosis (TB) and after treatment Clin Exp Immunol 1999 115 110 113 10.1046/j.1365-2249.1999.00783.x 9933428 PMC1905191

[B21] Kourbatova EV Leonard MK Jr Romero J Kraft C del Rio C Blumberg HM Risk factors for mortality among patients with extrapulmonary tuberculosis at an academic inner-city hospital in the US Eur J Epidemiol 2006 21 715 721 10.1007/s10654-006-9060-7 17072539

[B22] Kwara A Roahen-Harrison S Prystowsky E Kissinger R Adams R Mathison J Hyslop NE Manifestations and outcome of extra-pulmonary tuberculosis: impact of human immunodeficiency virus co-infection Int J Tuberc Lung Dis 2005 9 485 93 15875918

[B23] Al-Anazi KA Al-Jasser AM Evans DA Infections caused by mycobacterium tuberculosis in patients with hematological disorders and in recipients of hematopoietic stem cell transplant, a 12 year retrospective study Ann Clin Microbiol Antimicrob 2007 6 16 22 10.1186/1476-0711-6-16 18021401 PMC2200647

[B24] Lee DG Choi JH Kim YJ Lee S Min CK Kim DW Lee JW Min WS Shin WS Kim CC Hepatosplenic tuberculosis mimicking disseminated candidiasis in patients with acute leukemia Int J Hematol 2001 73 119 121 10.1007/BF02981913 11372747

[B25] Chakrabarti S Varma S Kochhar R Gupta S Gupta SK Rajwanshi A Hepatosplenic tuberculosis: a cause of persistent fever during recovery from prolonged neutropenia Int J Tuberc Lung Dis 1998 2 575 579 9661825

[B26] Andreu J Caceres J Pallisa E Martinez-Rodriguez M Radiological manifestations of pulmonary tuberculosis Eur J Radiol 2004 51 139 149 10.1016/j.ejrad.2004.03.009 15246519

[B27] Bodey GP Unusual presentations of infection in neutropenic patients Int J Antimicrob Agents 2000 16 93 95 10.1016/S0924-8579(00)00241-7 11053786

[B28] Mori T Leung CC Tuberculosis in the global aging population Infect Dis Clin North Am 2010 24 751 768 10.1016/j.idc.2010.04.011 20674802

[B29] Guidelines for the use of antimicrobial agents in patients with febrile neutropenia in Taiwan J Microbiol Immunol Infect 2005 38 455 457 16341349

[B30] Ahuja SS Ahuja SK Phelps KR Thelmo W Hill AR Hemodynamic confirmation of septic shock in disseminated tuberculosis Crit Care Med 1992 20 901 903 10.1097/00003246-199206000-00031 1597048

[B31] Jacob JT Mehta AK Leonard MK Acute forms of tuberculosis in adults Am J Med 2009 122 12 17 10.1016/j.amjmed.2008.09.018 19114163

